# Population pharmacokinetic modeling and clinical application of vancomycin in Chinese patients hospitalized in intensive care units

**DOI:** 10.1038/s41598-021-82312-2

**Published:** 2021-01-29

**Authors:** Zhong Lin, Dan-yang Chen, Yan-Wu Zhu, Zheng-li Jiang, Ke Cui, Sheng Zhang, Li-hua Chen

**Affiliations:** 1 Department of Clinical Pharmacy, Taizhou Hospital of Zhejiang Province Affiliated To Wenzhou Medical University, Ximen Street No. 150, Linhai, 317000 Zhejiang Province China; 2 Rehabilitation Department, Taizhou Hospital of Zhejiang Province Affiliated To Wenzhou Medical University, Xi Men Street No. 150, Linhai, 317000 Zhejiang Province China; 3 Intensive Care Unit, Taizhou Hospital of Zhejiang Province Affiliated To Wenzhou Medical University, Xi Men Street No. 150, Linhai, 317000 Zhejiang Province China; 4 Public Scientific Research Platform, Taizhou Hospital of Zhejiang Province Affiliated To Wenzhou Medical University, Xi Men Street No. 150, Linhai, 317000 Zhejiang Province China

**Keywords:** Drug delivery, Drug screening, Pharmacology

## Abstract

Management of vancomycin administration for intensive care units (ICU) patients remains a challenge. The aim of this study was to describe a population pharmacokinetic model of vancomycin for optimizing the dose regimen for ICU patients. We prospectively enrolled 466 vancomycin-treated patients hospitalized in the ICU, collected trough or approach peak blood samples of vancomycin and recorded corresponding clinical information from July 2015 to December 2017 at Tai Zhou Hospital of Zhejiang Province. The pharmacokinetics of vancomycin was analyzed by nonlinear mixed effects modeling with Kinetica software. Internal and external validation was evaluated by the maximum likelihood method. Then, the individual dosing regimens of the 92 patients hospitalized in the ICU whose steady state trough concentrations exceeded the target range (10–20 μg/ml) were adjusted by the Bayes feedback method. The final population pharmacokinetic model show that clearance rate (CL) of vancomycin will be raised under the conditions of dopamine combined treatment, severe burn status (Burn-S) and increased total body weight (TBW), but reduced under the conditions of increased serum creatinine (Cr) and continuous renal replacement therapy status; Meanwhile, the apparent distribution volume (V) of vancomycin will be enhanced under the terms of increased TBW, however decreased under the terms of increased age and Cr. The population pharmacokinetic parameters (CL and V) according to the final model were 3.16 (95%CI 2.83, 3.40) L/h and 60.71 (95%CI 53.15, 67.46). The mean absolute prediction error for external validation by the final model was 12.61% (95CI 8.77%, 16.45%). Finally, the prediction accuracy of 90.21% of the patients’ detected trough concentrations that were distributed in the target range of 10–20 μg/ml after dosing adjustment was found to be adequate. There is significant heterogeneity in the CL and V of vancomycin in ICU patients. The constructed model is sufficiently precise for the Bayesian dose prediction of vancomycin concentrations for the population of ICU Chinese patients.

## Introduction

Antibiotic therapy is often used for ICU patients for complicated infection treatment. More than 70% of Staphylococcus aureus isolates of ICU patients is methicillin resistant. Vancomycin has long been the gold standard for treating infections caused by this pathogen^1^. However, this antimicrobial is associated with several limitations, namely, its slow bactericidal activity, low penetration into certain tissues, high incidence of renal function damage and ototoxicity, increasing reports of resistance and failure, and potential minimum inhibitory concentration (MIC) ‘‘creep’’^2^.

To optimize the dosing of any antimicrobial agent, a firm understanding of the drug’s exposure-effect and exposure toxicity links are required^3^. American vancomycin guidelines suggested that AUC/MIC ratio ≥ 400 is the critical PK/PD target index for the optimal efficacy of vancomycin treatment. Therefore, individualized drug delivery is critical as there is considerable inter-patient (especially for ICU patients) variability in vancomycin exposure profiles in clinical practice. Compared with those of non-ICU patients, the extracellular fluid content or renal or liver function may be significantly modified in critically ill patients^4^. Some of these situations promote apparent changes in pharmacokinetic parameters in individual patients. Therefore, in the application of applicable standard dose regimens, failure of vancomycin therapy among ICU patients may further occur because of the inability of vancomycin to reach sufficient concentrations at the infection site^4^. Several studies have reported almost twice the mortality for critical patients infected by a pathogen not effectively treated with empirical antibiotic regimens^5^. The population pharmacokinetic (PPK) method is thought to be the most efficient method to improve vancomycin clinical therapeutic effectiveness and perform adverse reaction monitoring by precise dose adjustment^6^. Numerous specific population pharmacokinetic studies of vancomycin have been conducted that well describe the pharmacokinetic characteristics of specific populations of patients, such as those undergoing open heart surgery, extracorporeal membrane oxygenation therapy, high-volume hemofiltration, and critically ill patients et al^7,8^. However, the PPK models for Chinese ICU adult patients are still limited. In this paper, we constructed a PPK model of vancomycin for Chinese ICU adult patients from the random Real World with more comprehensive characteristic parameters of this subpopulation as variables. The aim was to identify covariates with a relevant influence on the pharmacokinetic parameters of vancomycin and to use this population pharmacokinetic model for the design of an individualized and accurate dosing schedule of vancomycin for ICU patients.

## Methods

This is a prospective, observational study carried out between July 2015 and December 2017 at the Tai Zhou Hospital of Zhejiang Province (466 patients hospitalized in the ICU, 294 for modeling, 80 for external validation and 92 for dose adjustment application verification), in which adult patients (> 18 years) were administered vancomycin antimicrobial treatment because of suspected or documented infection with MRSA. The dosage regimens of vancomycin (powder injection from Eli Lilly or Xinchang) were designed by experienced clinicians based on the patient's condition and renal function, including 0.5 g qd, 0.5 g q12h, 0.5 g q8h, 0.5 g q6h, 1 g qd, and 1 g q12h. Patients were excluded if they had received vancomycin therapy for < 7 days; were children or pregnant women; or were end-stage (end-stage renal disease, patients with terminal stage of tumor or hepatic failure). Data collected from patients’ medical records included age, total body weight, Sex, creatinine level, drug combination, and burn and continuous renal replacement therapy status (Burn-S, CRRT-S). Informed consent was obtained from the patients and their immediate relatives for this prospective and observational study. All the information that could identify the individual participants was hidden in the present paper. Ethical approval was obtained from the Ethics Committee of Tai Zhou Hospital of Zhejiang Province. All methods were performed in accordance with the relevant guidelines and regulations from the Ethics Committee of Tai Zhou Hospital of Zhejiang Province.

A total of 837 trough concentration (C_trough_) blood samples were collected 0.5 h before the next dose (after CRRT treatment for CRRT treatment patients), and 156 samples were collected at approach peak concentration (1 h after the peak concentration), both at the steady state (after the 6th dose) for most patients. All the samples (3–5 ml) were placed in 10 ml tubes containing coagulant and then centrifuged at 3000 rpm for 5 min. The supernatant serum was stored at − 80 °C for further testing.

Vancomycin serum concentrations were measured at the Clinical Pharmacokinetic Unit of Taizhou Hospital using HPLC and the chromatographic condition was similar with that we reported previously^9^, which was carried out on a Waters ODS2 reverse-phase column (250 mm × 4.6 mm, 5 μm particle size) with a mixture of acetonitrile, methanol and potassium dihydrogen phosphate buffer (0.05 M) (8:0.25:97.5, v/v) adjusted to pH 2.6 using phosphoric acid as the mobile phase. The multilevel calibration was 0.4–100.0 μg/ml with calibration line slope consistently > 0.999. The RSD of intra-day and inter-day assays were less than 7%, and the recovery rate was 95.5–103.2%. Serum creatinine was detected by the creatine oxidase method with a biochemistry analyzer (Beckmann CX5).

Kinetica, a nonlinear mixed-effects model population pharmacokinetic program, is practical and reliable software used for PPK analysis^10,11^. To our knowledge, no previous PPK studies have used Kinetica for vancomycin PPK model construction. In this study, we assessed the pharmacokinetic profile of vancomycin among ICU population patients with Kinetica version 4.4.1. As most of the trough concentrations of vancomycin was collected from sparse data at steady state, we fit the data with a one-compartment model to retrieve the pharmacokinetic parameters (CL and V).The covariate models and interindividual variability were expressed as following equation:

$$\upbeta _{i} = {\text{Z}}_{{\text{i}}}\upbeta + {\upeta }_{{\text{j}}} ,$$

where β_j_ denote as the parameter for the jth subject; Z_j_ is amatrix depending on the covariates Z_j_. When no covariates are included, β is the (p*1) vector of the mean parameters; η_j_ is the interindividual variability that is assumed normal distribution with mean 0 and covariance matrix C (p*p).

Population pharmacokinetic analysis was performed with 993 serum concentrations versus time from 374 adult patients (294 for model building and 80 for external validation, randomly selected by Kinetica), using a nonlinear mixed-effects modeling method with Kinetica. We reasonably fitted the plots of concentrations versus time with one-compartment models for most serum samples (n = 837) collected during the trough period (156 samples were collected during the approach peak period). An expectation–maximization iterative algorithm procedure (EM) was used for individualized pharmacokinetic parameter and model fitting. The initial pharmacokinetic parameters of V and CL were 52.14 L and 2.83 L/h as Ji et al. reported^6^. The basic model was fit only by EM procedure. The impact of the covariates on the pharmacokinetic parameters was investigated by a stepwise method. The final model was constructed by EM procedure with the screened covariates that have a significant influence on the pharmacokinetic parameters. The maximum likelihood evaluation (MLL) was used for internal and external model evaluation.

The EM algorithm, is an iterative procedure developed for finding maximum likelihood (ML) estimates for incomplete data. This is a two-step algorithm represented by E-step and M-step. The E-step is given current values of parameter estimates, to obtain the expectation of individual parameters, conditional on the observed data vector. The M-step is to obtain the ML posterior population mean and variance together with the residual error variance, given the individual parameter values. The iteration algorithm terminates continues when the relative change between two iterations for each of the estimated population parameters is lower than 1%.

Stepwise regression was used for the screened covariates. First, singer covariate was added to the basic model for check whether the included covariate had a significant effect on CL and V according to the results of the F-test. Then, the covariates with the potential influence CL and V were added sequentially and systematically according to the results of the F-test. The basic model contains only a constant term. The procedure selects for entry the covariate that produces the largest increase in F value in the presence of the previous covariate. The *P* value is calculated to check whether F-change is significant when the covariate is included (*P* < 0.05 represent that the added corresponding covariate to the model has a significant effect on CL and V, then the covariate should be included into the model; *P* > 0.05, delete the covariate). This process is continued until no more variables are admitted to the equation. The potential covariates to be screened included dopamine (1 if not used or 2 if used), creatinine (μmol/L), age (y), sex (1 if male or 2 if female), Vancomycin manufacturer (VM; 1 if made by Eli Lilly or 2 if by Xinchang), total body weight (kg), noradrenaline (NE; 1 if not used or 2 if used), continuous renal replacement therapy status (1 if treated by CRRT, 2 if not), burn status (1 if burn degree > 50%, acute convalescence or 2 if burn degree < 50%, 20 days after burn), furosemide (1 if not used or 2 if used) and dobutamine (1 if not used or 2 if used).

To compare two hierarchically related models (including internal validation and external validation) using the likelihood ratio test, the likelihood function was evaluated at the end of the algorithm, yielding the Akaike criterion (AIC), Schwartz criterion (BIC), and Log likelihood value (LL). Smaller values of AIC and BIC and larger values of LL represent better the prediction performance. Visual inspection of the distributions of the residual and weighted residual plots was also used for model evaluation. The concomitant vancomycin variables were screened with a stepwise method by Kinetica.

Bayes feedback method was used for external validation with random selected 80 patients from the included 374 patients. The mean prediction error (MPE) and mean absolute prediction error (MAE) represent the predicted precision of the final model. MPE and MAE calculated by the following equations:

$${\text{MPE}} = \frac{1}{{\text{n}}}\mathop \sum \limits_{{{\text{j}} = 1}}^{{\text{n}}} \left( {{\text{obs}}_{{\text{j}}} - {\text{calc}}_{{\text{j}}} } \right);\;\;{\text{MAE}} = \frac{1}{{\text{n}}}\mathop \sum \limits_{{{\text{j}} = 1}}^{{\text{n}}} \left| {{\text{obs}}_{{\text{j}}} - {\text{calc}}_{{\text{j}}} } \right|$$

where obs_j_ and calc_j_ denote as the observed and predicted vancomycin concentrations for the j^th^ subject, respectively.

The individual pharmacokinetic parameters (CL and V) of 92 patients hospitalized in the ICU whose routine clinical monitoring steady-state trough concentrations (more than 6 dose) exceeded the target range (10–20 μg/ml) were predicted by one point Bayes feedback method with the constructed final model and used for individualized drug delivery design. The target trough concentration was set at the range of 10–20 μg/ml that was recommended by the Vancomycin Clinical Application of Chinese Expert Consensus (2012 edition). After the 6th adjust dose, the plasma samples of those patients were collected at the trough concentration period and detected for verified the predicted precision of Bayes feedback method with the final model.

## Results

### Patients’ demographic data and clinical features

As shown in Table 1, 374 adult patients (male, n = 254; female, n = 120) were infected with MRSA or G^+^ bacteremia or were suspected of G^+^ bacteremia infection. The median of the age was 62 years, and the age range was 18–93 years. Vancomycin was manufactured by Eli Lilly (American, 61.2%) and Xinchang (Chinese, 38.8%). The infection sites were highly varied: 18.2% pulmonary, 55.6% blood, 11.2% skin soft tissue, 6.4% abdominal and 8.6% others.

**Table 1:** Summary of patient characteristics.

Parameters	Value (n = 374)
Age (y)	62.0 (18.0,93.0)
TBW (kg)	65.0 (40.0,90.6)
**Sex (n)**	
Male	254 (67.9%)
Female	120 (32.1%)
**VM (n)**	
Eli Lilly	229 (61.2%)
Xinchang	145 (38.8%)
**CRRT-S (n)**	
No CRRT	287 (76.7%)
CRRT	87 (23.3%)
**Burn-S (n)**	
No burn	342 (91.4%)
Burn	32 (8.6%)
Cr (μmol/L)	71.0 (28.0,581.0)
**Infection site**	
Pulmonary	68 (18.2%)
Blood	208 (55.6%)
Soft tissue	42 (11.2%)
Abdominal	24 (6.4%)
Other	32 (8.6%)
C_trough_	16.3 ± 12.4
C_appro peak_	36.0 ± 19.4

C_trough_ means trough concentration that of blood samples collected 0.5 h before the next dose; C_appro peak_ means approach peak concentration that of the blood samples collected 1 h after completion of drug infusion.

TBW, body weight; VM, Vancomycin manufacture; Burn-S, Burn status; CRRT-S, continuous renal replacement therapy status; Cr, creatinine;

### Population pharmacokinetic analysis

In Table 2, we summarize the covariates that significantly influence vancomycin disposition via one-by-one inclusion in the basic model (the covariates of sex, furosemide and dobutamine have not been displayed in Table 2 as they were uncorrelated with CL and V). Table 3 and 4 summarize how the covariates of DA, Cr, Burn-S, TBW and CRRT-S (*P* < 0.05) and Cr, Age and TBW (*P* < 0.05) were retained in the final model for CL and V, respectively, by including them all in the basic model, yielding the final covariate equations: CL = 0.78 + 0.036*DA − 0.007*Cr − 0.43*Burn-S + 0.067*TBW + 0.41*CRRT-S, V = 46.47 − 0.04*Cr − 0.07*Age + 0.16*TBW. The PPK parameters of V and CL obtained by the final model were 60.71 L and 3.16 L/h.

**Table 2:** Intermediate models showing the influence of the different covariates on the vancomycin pharmacokinetic parameters.

	Model	F change
Basic model	CL = θ_1_ V = θ_2_	/
**Covariates forCL**		
DA	CL = θ_1_ + θ_2_*DA V = θ_3_	5.3 (*P* = 0.022)
Cr	CL = θ_1_ + θ_2_* Cr V = θ_3_	93.9 (*P* = 0.000)
VM	CL = θ_1_ + θ_2_* VM V = θ_3_	3.7 (*P* = 0.041)
Burn-S	CL = θ_1_ + θ_2_* Burn-S V = θ_3_	9.1 (*P* = 0.003)
TBW	CL = θ_1_ + θ_2_* TBW V = θ_3_	116.4 (*P* = 0.000)
CRRT-S	CL = θ_1_ + θ_2_* TBW V = θ_3_	27.9 (*P* = 0.000)
**Covariates forV**		
Cr	V = θ_1_ + θ_2_* Cr CL = θ_3_	104.4 (*P* = 0.000)
Age	V = θ_1_ + θ_2_* Cr CL = θ_3_	56.7 (*P* = 0.000)
VM	CL = θ_1_ + θ_2_* VM V = θ_3_	3.9 (*P* = 0.043)
NE	CL = θ_1_ + θ_2_* NE V = θ_3_	4.0 (*P* = 0.043)
TBW	V = θ_1_ + θ_2_* TBW CL = θ_3_	93.8 (*P* = 0.000)
CRRT-S	V = θ_1_ + θ_2_* Cr CL = θ_3_	28.8 (*P* = 0.000)

Increases in the F value (F-change) represent the degree of influence of the addition of the corresponding covariate on CL and V (*P* < 0.05 represent that addition of the corresponding covariate to the base model has a significant effect on CL and V).

*DA* dopamine; *Cr* serum creatinine; *VM* vancomycin manufacturer; *Burn-S* burn status; *TBW* total body weight; *CRRT-S* continuous renal replacement therapy status; *NE* noradrenaline.

**Table 3:** Covariates for CL screened by the stepwise method.

Variable	F Test	F-change	*P* value
DA	/	5.3	0.022
DA, Cr	82.0	156.6	0.000
DA, Cr, VM	54.6	0.1	0.712
DA, Cr, Burn-S	56.4	3.9	0.050
DA, Cr, Burn-S, TBW	109.6	185.1	0.000
DA, Cr, Burn-S, TBW, CRRT-S	90.4	6.6	0.010
Final covariable equation: CL = 0.78 + 0.036*DA − 0.007*Cr − 0.43*Burn-S + 0.067*TBW + 0.41*CRRT-S			

Increases in the F value (F-change) represent the degree of influence of the addition of the corresponding covariate on CL (*P* < 0.05 retained the corresponding covariate; *P* > 0.05 deleted the corresponding covariate).

**Table 4:** Covariates for V screened by the stepwise method.

Variable	F Test	F-change	*P* value
Cr	/	104.4	0.000
Cr, VM	53.1	1.6	0.210
Cr, age	66.5	22.5	0.000
Cr, age, NE	44.2	0.02	0.876
Cr, age, TBW	55.9	25.8	0.000
Cr, age, TBW, CRRT-S	41.9	0.3	0.563
Final covariable equation: V = 46.47 − 0.04* Cr − 0.07*Age + 0.16*TBW			

Increases in the F value (F-change) represent the degree of influence of the addition of the corresponding covariate on V. (*P* < 0.05 retained the corresponding covariate; *P* > 0.05 deleted the corresponding covariate).

**Figure 1:**
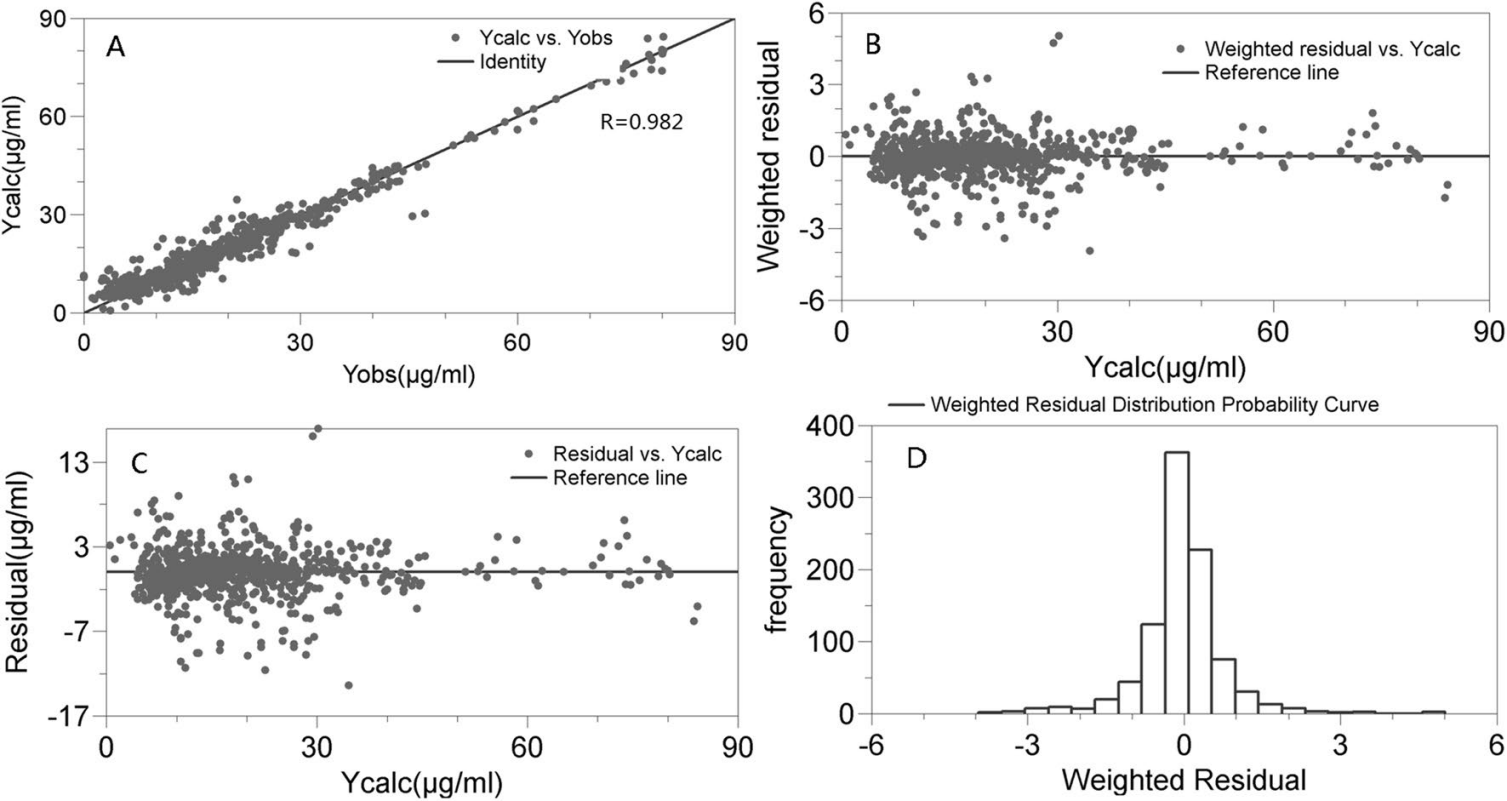
Goodness-of-fit plots of the final model for vancomycin. (**A**) Individual predictions of vancomycin versus observed concentrations. (**B**) Individual predictions of vancomycin versus predicted weighted residuals. (**C**) Individual predictions of vancomycin versus predicted residuals. (**D**) Gaussian distribution of weighted residuals.

The model evaluation parameters of AIC (3.16 versus 3.36), BIC (3.17 versus 3.36) and LL (− 2922 versus − 3121) from the final model and the basic model indicated that the goodness-of-fit performance of the final model was superior to that of the basic model. The goodness-of-fit plots in Fig. 1 also show that the vancomycin concentration predicted by the final model adequately described the observed vancomycin concentrations. The relationship between the predicted values and observed values of the vancomycin concentration was well approximated by the line y = x (Fig. 1A), and the weighed residuals (Fig. 1B) and the residuals (Fig. 1C) of the estimated drug concentrations were distributed around the line y = 0. Furthermore, the weighted residuals (Fig. 1D), V (not provided) and CL (not provided) were normally distributed.

**Figure 2:**
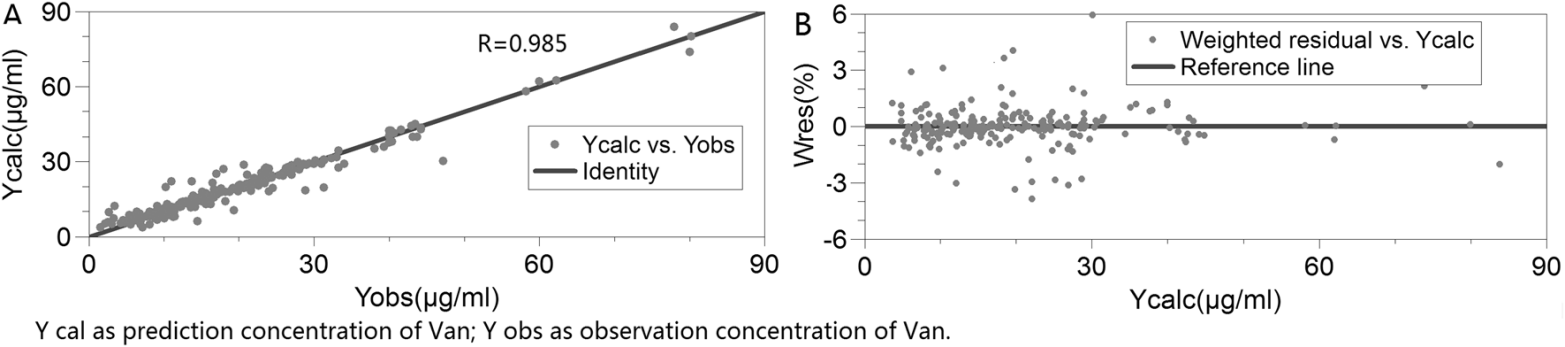
Goodness-of-fit plots of external validation. (**A**) Individual predictions of vancomycin versus observed concentrations. (**B**) Individual predictions of vancomycin versus predicted weighted residuals.

The relationship between the observed concentrations and the individual predicted values from the external validation data were closely approximated by the line y = x (Fig. 2A), and the weighted residuals were randomly and homogenously distributed around the line y = 0 (Fig. 2B). As Table 5 shows, the mean prediction error (MPE, 4.76%) and mean absolute prediction error (MAE, 12.61%) of the final model were also much better than those of the initial model (MPE, − 10.11%; MAE, 18.17%) in the external validation process according to the Bayesian feedback method.

**Table 5:** External evaluation parameters of the basic model and final model.

Parameters	Final model Mean (95%CI)	Basic model
MPE (%)	4.76 (0.61, 8.90)	− 10.11 (− 15.15, − 5.07)
MAE (%)	12.61 (8.77,16.45)	18.17 (13.55, 22.79)

*MPE* mean prediction error; *MAE* mean absolute prediction error.

Table 6 summarized the results of the basic and final population models. All the parameters of the final were reliably predicted, since the percentage of relative standard estimated error was less than 40%.The population coefficient of variation decreased from 53.27% and 43.15% to 41.72% and 35.14% from the basic to the final model for CL and V, respectively. The individualized heterogeneity increased from 25.72% and 37.31% to 56.01% and 55.19% from the basic to the final model for CL and V.

**Table 6:** Summary of results for the basic and final population models.

Parameter		95%CI
**Basic model**		
CL (L/h) (RSE%)	2.63 (25.72)	2.43,2.85
%CV of CL	53.27%	/
RSEE% of CL	38.23	/
V (L) (RSE%)	55.32 (27.31)	52.32,58.21
%CV of V	43.15%	/
RSEE% of V	21.36	/
MAE (%)	32.62	23.61, 40.31
**Final model**		
CL (L/h) (RSE%)	3.16 (56.01)	2.83, 3.40
%CV of CL	41.72%	/
θ_1_ (RSEE%)	0.78 (33.46)	/
θ_2_ (RSEE%)	0.036 (36.15)	/
θ_3_ (RSEE%)	0.007 (32.32)	/
θ_4_ (RSEE%)	0.43 (36.51)	/
θ_5_ (RSEE%)	0.067 (22.15)	/
θ_6_ (RSEE%)	0.41 (35.65)	/
V (l^−1^) (RSE%)	60.71(55.19)	53.15, 67.46
%CV of V	35.14%	/
θ_7_ (RSEE%)	46.47 (36.21)	/
θ_8_ (RSEE%)	0.04 (32.43)	/
θ_9_ (RSEE%)	0.07 (38.17)	/
θ_10_ (RSEE%)	0.16 (32.63)	/
MAE (%)	8.71	5.73, 19.23

%CV as coefficient of variation; RSEE% as relative standard prediction error; θ as regression parameter; RSE% as relative standard error.

**Figure 3:**
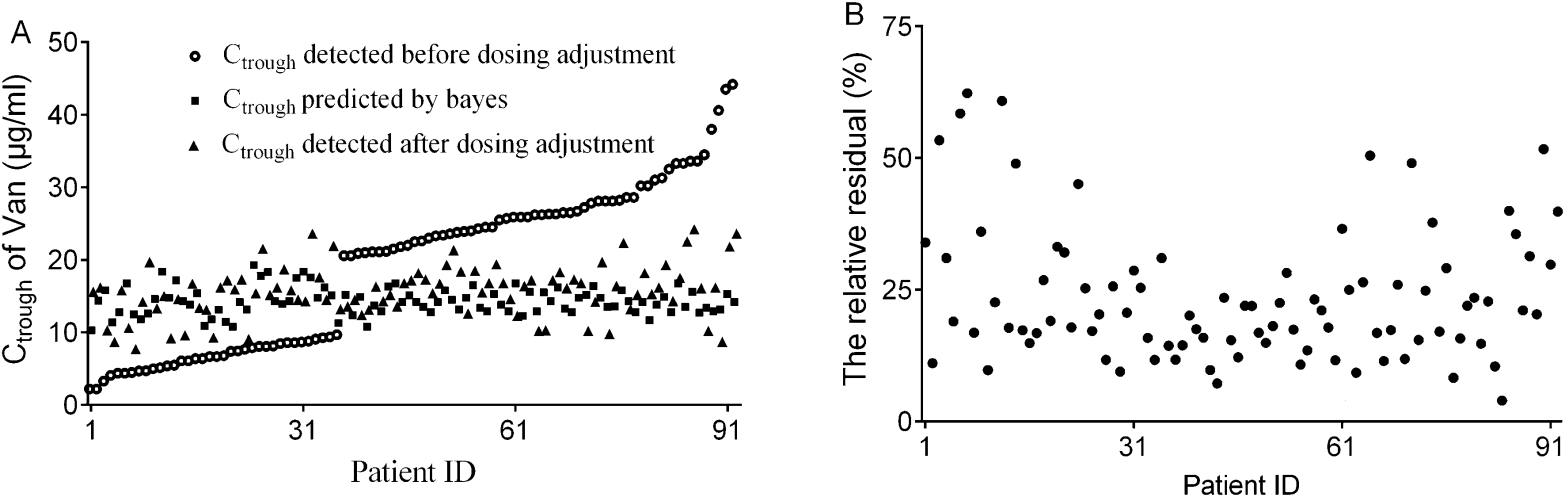
Results of dosing adjustment for 92 patients hospitalized in the ICU by Bayes feedback based on the final model. (**A**) The distribution diagram of routine C_trough_ detected before dosing adjustment, C_trough_ predicted by the Bayes method and C_trough_ detected after dosing adjustment. (**B**) The relative predicted residuals of C_trough_ predicted by the Bayes method versus C_trough_ detected after dosing adjustment.

As Fig. 3A shows, of the 92 ICU patients whose C_trough_ values were outside the target range (10–20 µg/ml), 90.2% of their detected C_trough_ were distributed within the target range after dosing adjustment and were close to the distributed location of the C_trough_ values predicted by the Bayes method for the 92 patients hospitalized in ICU, indicating that their vancomycin dosage regimens should be individually adjusted due to inappropriate serum drug concentration on routine monitoring. The prediction accuracy was also good, as Fig. 3B shows that most of the predicted residuals were below 40%, the mean value of the predicted residuals was 23.54% with the 95% confidence interval of 20.92–26.17%.

Our study design indicates that the covariates of general ICU patients, rather than those of specific populations, are influencing factors for vancomycin pharmacokinetic parameters. This is in agreement with other adult population studies published for vancomycin, as shown in Table 7, where Cr, age and weight are listed as common covariates that influence the disposition of vancomycin.

**Table 7:** Vancomycin PPK model studies in adult patients by the nonlinear mixed-effects modeling method.

Study	patient n/type	Final model of CL, V	CV% of CL, V	MAE (%)
This study	374; ICU	CL =0.78 + 0.036*DA − 0.007*Cr -0.43* Burn-S + 0.067*TBW + 0.41* CRRT-S	41.7%	11.7
			35.1%	
		V = 46.47 − 0.04*Cr − 0.07*Age + 0.16* TBW		
Natalia^1^	191; ICU	CL(ml·min^−1^·kg^−1^) = 0.67*CL_Cr_ (ml·min^−1^ ·kg^−1^) + AGE^−0.24^	17.8%	9.5
			49.4%	
		V (L·kg^−1^) = 0.82*2.49^A^, A = 0 or 1 if Cr ≤ 88.4 or Cr > 88.4 μmol/L		
Llopis^12^	30; ICU	CL(L·h^−1^) = 0.034*CL_Cr_(ml·min^−1^) + 0.015* TBW	29.2%	23.9
		Vc (L) = 0.414* TBW	36.4%	
		Vp (L) = 1.32*TBW	39.8%	
Christine^13^	102; unstable renal function	CL(L·h^−1^) = 2.97*(1 + 0.0205*(CL_Cr_ − CL_CrMedian_))	27%	15
		V (L·kg^−1^) = 1.24	36%	
Yasuhara^14^	190;Adult Japanese	CL(L·h^−1^) = 0.0487*CL_Cr_(ml·min^−1^),	38.5%	23.7
		if CL_Cr_ < 85; CL(L·h^−1^) = 3.51, if CL_Cr_ > 85	25.4%	
		V = 60.71		

*Vc* central compartment volume; *Vp* peripheral compartment volume; *%CV* as coefficient of variation; *MAE* as mean absolute prediction error.

## Discussion

Vancomycin has been used to treat infections caused by Staphylococcus aureus isolates for decades, especially as part of anti-MRSA treatment^15^. However, vancomycin is a potentially toxic drug; High doses have a high rate of nephrotoxicity and ototoxicity, and a low dose can reduce the effectiveness of the therapy and increase the propensity to bacterial resistance^16^. Many papers report that the vancomycin PPK model can fit the pharmacokinetic parameters of specific populations of patients well and simulate an improved dosage regimen with NONMEM^7,17,18^. However, models with large sample sizes and complete variables representative of the features of Real World ICU patients are limited, as the pharmacokinetic parameters and serum drug concentration levels of these patients are much more complicated than those of normal patients^19,20^.Therefore, the aim of this study was to develop a PPK model of vancomycin in the identified subpopulation of Chinese ICU patients with severe pathological features by screening potential conventional and representative covariate factor-related pharmacokinetic parameters that can be used for individualizing drug regimens for these patients by a Bayesian feedback method.

The mean values of V (60.71 L) and CL (3.16 L/h) obtained with the final model by Kinetica in this study is similar to that of other studies, which have reported values of 31.24–91.76 L for V and 2.89–5.93 L/h for CL in non-ICU patients^7,21^. Our final population model yielded comparable results for coefficient of variation (CV%) and individual heterogeneity on CL (41.7%, 56.01%) and V (35.1%, 55.19%), which are significantly higher than those typically seen in patient populations not admitted to the ICU (Table 6), probably due to the effect of patient pathophysiological status on drug disposition and the pronounced heterogeneity of critically ill populations in the ICU^22^.

In the present study, we revealed that the CL would be accelerated in the presence of rescue drugs (DA), which has not been reported in other vancomycin PPK model studies, as shown in Table 7. However, this phenomenon has been discussed in a previous study on other drugs, which indicate that dopamine and furosemide could lead to the increased CL level of levofloxacin observed in some patients hospitalized in the neurosurgical emergency room during treatment of ventilator-associated pneumonia with levofloxacin 500 mg twice daily^23^. For an adequate sample size of burned patients (n = 32), we also detected that burn status increased the CL. In fact, from a pharmacokinetic aspect, different pathophysiological changes occur in burn patients, especially at the acute convalescence phase, which is common characterized by an increased level of cardiac output that results in elevated renal blood flow and in turn glomerular filtration rates. Kateřina noted that increased Cr levels are a distinct marker for renal dysfunction^24^. In our study, the level of Cr was also negatively correlated with the renal clearance rate of vancomycin, which corresponds with other studies^7,8,25^. Obesity is related to numerous physiological adaptations, which involve increased kidney mass, renal blood flow, creatinine and vancomycin clearance rate^19^. As our final model shows, an increased TBW also increases the level of vancomycin clearance rate. CRRT is widely used for treating severe acute kidney injury patients^26,27^. Previous studies have reported that CRRT accelerates the excretion of vancomycin^28,29^, a result that contradicts ours. The key factor explaining this was not confirmed but may reflect variable patient factors and CRRT techniques^30^.

As the final model in Table 4 shows, the apparent distribution volume of vancomycin were associated with age and TBW, which is consistent with studies in other patient populations^7,8^. As is well known, the tissue distribution capacity of vancomycin is enhanced in patient populations with greater TBW^31^. The underlying mechanism explaining the decreased V for patients with higher age could be attributed to impaired renal function and changes in tissue composition, which is consistent with previous studies^21^. Previous investigation have documented increases in V of aminoglycosides in critical patients on the basis of changes in the body compartments due to fluid overload in attempts to maintain haemodynamics^32^. In addition, Nataliafirst reported that the vancomycin V correlate with the renal function, in which the V of the patients with Cr > 88.4 μmol/l increased more than twofold than in the patients with a Cr value below this threshold (148.19 L vs. 59.86 L)^1^. This result was contradiction with our final model described the negative correlation between V and Cr. This discrepant phenomenon generated may be attributed to the sample heterogeneity, for 85.7% of our population ICU patients with renal dysfunction were elderly patients (> 70 years) with a significant decreased TBW compared to the whole inclusion group (46.6 kg vs. 65.0 kg). Further research is needed to clarify the precise relation between vancomycin V and Cr in critical patients.

There are growing clinical studies, in spite of mostly retrospective in essence, supported that AUC/MIC ratio ≥ 400 for vancomycin treatment is the important PK/PD target index for the optimal efficacy of the patients infected with MRSA bloodstream^33,34^. However, calculation of AUC in clinical practice involved collection of multiple vancomycin serum concentrations during the same dosing interval, with subsequent use of PK software that was not readily available at all institutions. As such, American expert guidelines recommended monitoring trough concentrations between 15 and 20 mg/L for serious infections as alternative index for the AUC/MIC ratio are more practical based on the historical difficulty in estimating the AUC in clinical practice^35^. The Vancomycin Clinical Application of Chinese Expert Consensus also recommended that the trough concentrations of vancomycin should be maintained between 10 and 20 mg/L for optimizing the efficacy and reducing toxic effects^36^. Thus, we used this concentration range as the target value to design the adjustment dosage regimen for these patients whose clinical monitoring steady state trough concentrations outrange 10–20 mg/L. As the result shown in Fig. 3, the individual pharmacokinetic parameters of Chinese ICU patients can be effectively calculated by Bayes feedback method with the final model in kinetica with one point steady C_trough_. The ultimate predicted accuracy for the application data set was inferior to that of the external evaluation data set (23.54% vs 8.71%) because of the unstable profile of pharmacokinetics in ICU patients. Further, this model can be implemented into other Bayes dosing software that can be utilized for individualized drug delivery design on Chinese ICU patients receiving vancomycin.

This study has several limitations that should be considered regarding interpretation. Firstly, this is not a randomized study, thus greater heterogeneity were existed in different patients that would influence the accuracy of population prediction, despite it is excellent in predicting individualized pharmacokinetic parameters with one point C_trough_ by Bayes method. Secondly, the patients included only from a single center, further investigations should be implemented to validate our models before extrapolating our observations. In addition, the relationship between AUC and the clinical outcomes also should be evaluated with this prospective data in the future study because the guideline viewed that the vancomycin exposure index AUC is current still be as the most effective target for treatment maximal effect and minimal toxicity.

In conclusion, vancomycin PPK models specifically designed for ICU patients were established and evaluated in this study. Even though the final models will be useful for initial dosage selection and Bayesian feedback prediction for the individualized pharmacokinetic parameters of ICU patients, further study should focus on these pharmacokinetic parameters and the therapeutic effect of the selected dosages to determine their relationship in clinical practice.
